# Proteomic Analysis of Dysfunctional Liver Sinusoidal Endothelial Cells Reveals Substantial Differences in Most Common Experimental Models of Chronic Liver Diseases

**DOI:** 10.3390/ijms241511904

**Published:** 2023-07-25

**Authors:** Mar Gil, Mikel Azkargorta, Carla Fuster, María Martínez-Gómez, Imma Raurell, Aurora Barberá, Juan Manuel Pericàs, Diana Hide, Felix Elortza, Joan Genescà, María Martell

**Affiliations:** 1 Liver Diseases, Vall d’Hebron Institut de Recerca (VHIR), Liver Unit, Hospital Universitari Vall d’Hebron (HUVH), Vall d’Hebron Barcelona Hospital Campus, Universitat Autònoma de Barcelona (UAB), 08035 Barcelona, Spain; mar.gilsoler@gmail.com (M.G.); carlafuster.ruiz@gmail.com (C.F.); maria.martinez@vhir.org (M.M.-G.); imma.raurell@vhir.org (I.R.); aubarbel@gmail.com (A.B.); juanmanuel.pericas@vallhebron.cat (J.M.P.); dianahide@gmail.com (D.H.); 2 Proteomics Platform, CIC bioGUNE, BRTA (Basque Research & Technology Alliance), Bizkaia Science and Technology Park, 48160 Derio, Spain; mazkargorta@cicbiogune.es (M.A.); felortza@cicbiogune.es (F.E.); 3 Centro de Investigación Biomédica en Red de Enfermedades Hepáticas y Digestivas (CIBERehd), 28029 Madrid, Spain

**Keywords:** chronic liver disease, liver sinusoidal endothelial cells, endothelial dysfunction, proteomics, animal models

## Abstract

Molecular markers of dedifferentiation of dysfunctional liver sinusoidal endothelial cells (LSEC) have not been fully elucidated. We aimed at deciphering the molecular profile of dysfunctional LSEC in different pathological scenarios. Flow cytometry was used to sort CD11b^−^/CD32b^+^ and CD11b^−^/CD32b^−^ LSEC from three rat models of liver disease (bile duct ligation-BDL; inhaled carbon tetrachloride-CCl4; and high fat glucose/fructose diet-HFGFD). A full proteomic profile was performed applying nano-scale liquid chromatography tandem mass spectrometry (nLC-MS) and analyzed with PEAKS software. The percentage of CD32b^−^ LSEC varied across groups, suggesting different capillarization processes. Both CD32^+^ and CD32b^−^ LSEC from models are different from control LSEC, but differently expressed proteins in CD32b^−^ LSEC are significantly higher. Heatmaps evidenced specific protein expression patterns for each model. Analysis of biological significance comparing dysfunctional CD32b^−^ LSEC with specialized CD32b^+^ LSEC from controls showed central similarities represented by 45 common down-regulated proteins involved in the suppression of the endocytic machinery and 63 common up-regulated proteins associated with the actin-dependent cytoskeleton reorganization. In summary; substantial differences but also similarities in dysfunctional LSEC from the three most common models of liver disease were found, supporting the idea that LSEC may harbor different protein expression profiles according to the etiology or disease stage.

## 1. Introduction

Liver cirrhosis is the final stage of progressive chronic liver disease (CLD) in which fibrosis and nodular distribution of the liver parenchyma occur, causing organic dysfunction, liver failure and death due to a variety of etiologies [1]. Hepatic steatosis, excessive alcohol consumption and viral hepatitis infection are the most frequent causes of CLD nowadays [2]. Portal hypertension is a complication of advanced CLD and in turn one of the main drivers of its progression. The primary factor in the development of portal hypertension is a marked increase in intrahepatic vascular resistance (IHVR) caused by the mechanical consequences of the disruption of the liver vascular architecture together with an increased hepatic vascular tone. This dynamic component is strongly related to alterations in liver sinusoidal endothelial cells (LSEC) that, upon chronic injury, exhibit an imbalance of vasoactive molecules favoring vasoconstriction and increasing IHVR [3].

LSEC are highly specialized endothelial cells that interface blood components and form the barrier of liver sinusoids, being pivotal regulators of the hepatic microcirculation with a key role in sinusoidal crosstalk [4,5]. LSEC constitute a permeable barrier due to the presence of fenestrae and the absence of a basement membrane, which allows direct communication between sinusoidal blood and the subendothelial space of Disse [6,7,8,9]. This highly specialized phenotype includes the expression of specific surface markers, like the CD32b surface antigen, which correlates with the presence of fenestrae [10]. CD32b, the only Fc gamma-receptor expressed by LSEC, is highly expressed in these cells and enables efficient endocytosis of small soluble IgG-antigen complexes [7] via clathrin-mediated endocytosis [11]. In this sense, one of the major functions of LSEC is the elimination of macromolecules and small particulates from the blood thanks to their active endocytosis receptors, making LSEC the body’s most effective scavenger cells [11,12]. Under physiological conditions, LSEC are responsible for maintaining hepatic homeostasis, metabolite transport and vascular tone, facilitating oxygenation of hepatocytes and enhancing hepatocytes’ exposure to macromolecules from the portal circulation. LSEC possess anti-inflammatory and anti-fibrogenic properties by preventing the activation of Kupffer cells and hepatic stellate cells. However, under pathological conditions, LSEC de-differentiate, losing fenestrae properties and specific markers like CD32b, in a process known as capillarization. Complete loss of fenestrae seems to precede the development of most CLD [13,14] and involves reversion to a common vascular endothelial phenotype and loss of specialized functions.

The rationale of this study is based on (i) recognizing LSEC as the first inducers of liver damage, (ii) the loss of CD32b receptor as a hallmark of capillarization and (iii) the belief that a therapy specifically aimed at CD32b^−^ LSEC could stop or slow down the pathological process. Although specific markers of LSEC under physiological conditions have been described in the literature [7,11,12], there are no specific markers identified that are expressed exclusively/differentially in dysfunctional LSEC. Moreover, patients suffering from diverse liver diseases at different stages will have a dissimilar proportion of dysfunctional LSEC at various degrees of de-differentiation and harboring particular protein profiles. 

On the other hand, animal experimental models have proven thus far as the most useful tool to test new therapies in CLD. Despite some disadvantages, e.g., no single model covers the entire spectrum of human liver dysfunction and in some cases the translation from the animal to drug development in humans is not straightforward, each particular model is useful to understand specific aspects of the disease, with certain models being more adequate to tackle particular etiologies or stages of CLD [15]. 

Herein, we aimed to decipher the molecular profile of dysfunctional LSEC through differential protein expression profiling of healthy CD32^+^ LSEC vs. capillarized dysfunctional CD32b^−^ LSEC obtained from various experimental models of liver disease in the rat representing different pathological situations in order to improve the effectiveness of potential therapeutic agents.

## 2. Results

### 2.1. LSEC Sorting 

Table 1 summarizes the rat models from which LSEC were isolated from the whole liver and sorted, along with their sorting results. Sorting by flow cytometry of CD32b^+^ and CD32b^−^ LSEC subtypes yielded different numbers of cells depending on the animal model. Comparing the mean percentage of CD32b^−^ LSEC identified in BDL (44.6%) and HFGFD (16.9%) with their CTRL-SD (4.5%), and both models showed an increase in CD32b^−^ cells, while the mean percentage of CD32b^−^ LSEC from the CCl_4_ model (6%) showed no differences from its CTRL-W (5.7%).

### 2.2. Differential Proteomic Expression

A total of 60 samples (from 30 final rats) of both sorted CD32b^+^ and CD32b^−^ LSEC, containing at least 10^4^ cells each, from our three models (BDL, HFGFD and CCl_4_) and from their respective controls (CTRL·SD and CTRL·W) were analyzed at the Proteomics platform of CIC-BioGUNE to identify differentially expressed proteins in the three models of liver diseases. The proteomic study allowed the identification of 2.016 proteins, all qualified with 2 or more peptides, and 1.891 of those qualified with 2 or more unique peptides at a false discovery rate (FDR) < 1%.

**Figure 1:**
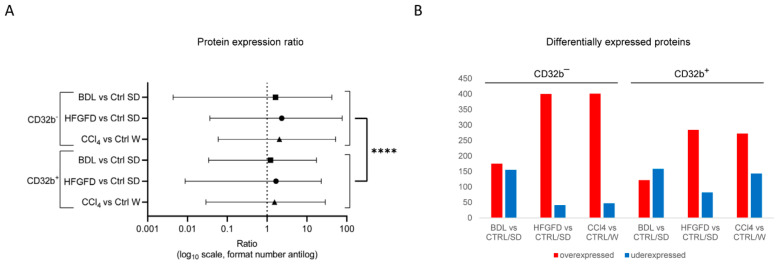

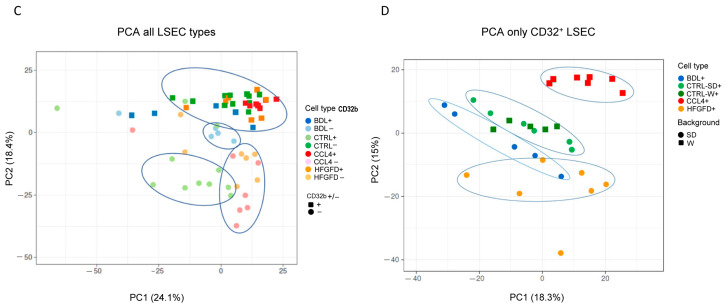
Protein expression induced by each model. (**A**) Representation of the mean and range of the expression ratio in all detected proteins. Mean is marked with the symbol, and range represented with the line. The x axis is on a logarithmic (log10) scale, with the numbers in antilog format. (**B**) Representation of the number of proteins detected that are differentially expressed (ratio ≥ 1.5 or ≤0.67, *p* < 0.05) between the groups of each comparison. (**C**) PCA performed with all sorted LSEC. Black squares and circles denote CD32b positive or CD32b negative LSEC, respectively. (**D**) Further analysis using only CD32b^+^ LSEC. Black circles and squares denote Sprague-Dawley or Wistar background, respectively. LSEC, liver sinusoidal endothelial cells; BDL, bile duct ligation model; HFGFD, high fat glucose fructose diet model; CCl_4_, carbon tetrachloride model; CTRL, control; SD, Sprague-Dawley; W, Wistar; **** *p* < 0.0001.

We first compared each LSEC subtype (CD32b^+^ and CD32b^−^) generated in the three models with their respective counterpart in the healthy control animals, in order to analyze (i) how positive and negative cells changed during the capillarization process in each case, (ii) which subset of cells (CD32b^+^ and CD32b^−^) had experienced more prominent changes and (iii) to be sure that in the case of the CD32b^−^ from the CCl_4_ model, we were dealing with de-differentiated LSEC, different from the CD32b^−^ cells present in the healthy rats. Figure 1A represents a general view of the ratio data from all the identified proteins, showing the mean and range of each comparison through a logarithmic scale that represents, symmetrically, under- and over- expressed ratio values. Comparisons between CD32b^−^ LSEC groups appeared to have ratio means further on the right compared with those between CD32b^+^ LSEC, showing that CD32b^−^ LSEC samples from the three models had a higher number of differentially over-expressed proteins compared to their respective controls than CD32b^+^ LSEC samples, which were more alike to their corresponding CD32b^+^ LSEC controls. It is worth noting that although the CCl_4_ model generated no significant increments in CD32b^−^ LSEC cells (Table 1), these cells were indeed very different from their controls, as they showed the highest number of differentially expressed proteins, suggesting differences in the capillarization process of the three models (Figure 1B).

In accordance with the observation that CD32b^−^ LSEC experienced a more pronounced change than CD32b^+^ during the alteration induced by the three models, principal component analysis (PCA) made with all 60 samples revealed also that CD32b^+^ LSEC were more similar between disease models and healthy controls (clustering closely together) than to CD32b^−^ LSEC of their respective model (clustering in separate groups from their control) (Figure 1C). Surprisingly, and despite their different backgrounds, CD32b^−^ LSEC from HFGFD and CCl_4_ models clustered together and not with those from the BDL model. Nevertheless, further PCA, including only CD32b^+^ LSEC, also grouped them in separate groups corresponding to each model (Figure 1D), representing protein expression differences, whereas positive control cells from both Sprague-Dawley and Wistar backgrounds clustered together and separately from models.

**Figure 2:**
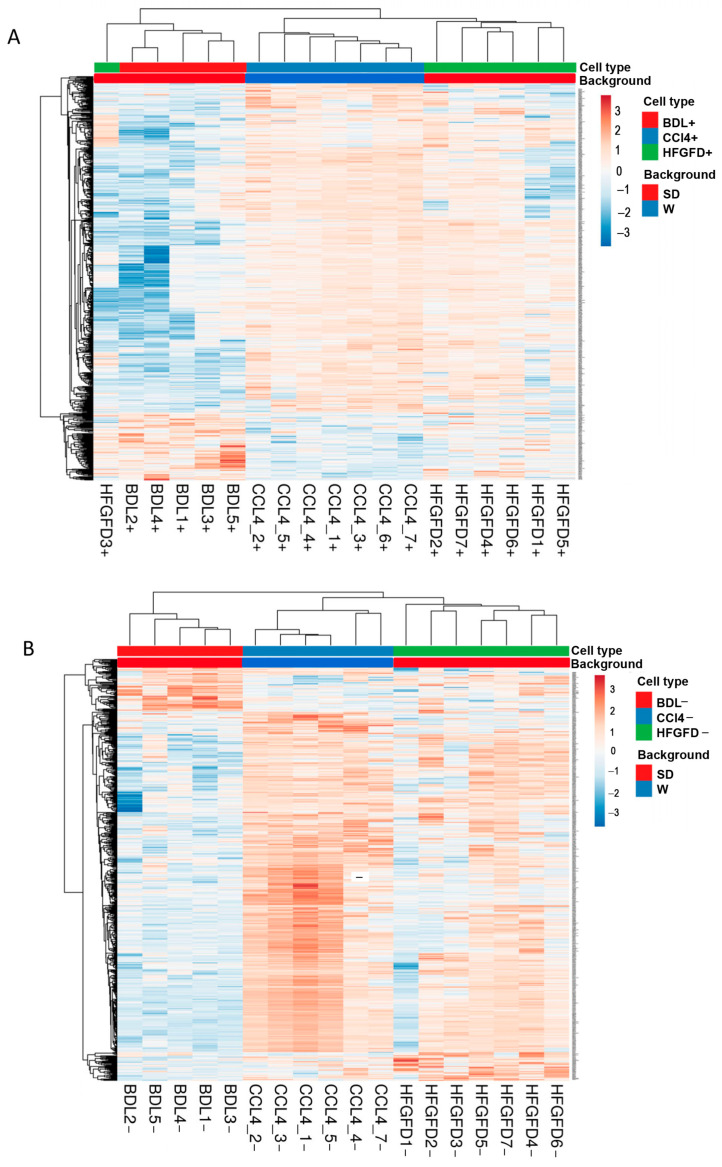
Changes in LSEC subtypes generated in the three models compared with their respective counterparts in the healthy controls. Expression profile heatmaps of differentially expressed proteins in (**A**) CD32b^+^ LSEC and (**B**) CD32b^−^ LSEC. BDL, bile duct ligation model; HFGFD, high fat glucose fructose diet model; CCl_4_, carbon tetrachloride model; SD, Sprague-Dawley; W, Wistar; LSEC, liver sinusoidal endothelial cells.

To better outline the differences between groups, we compared the differentially expressed proteins between models (BDL vs. HFGFD vs. CCl4) by a supervised ANOVA test. Using the normalized abundance values for analysis and plotting, heatmaps respectively containing 632 and 643 proteins for positive and negative CD32b LSEC were obtained (Figure 2). These heatmaps yielded notably different patterns for each of the three models, with the HFGFD and CCl4 models being again more similar to each other than to the BDL model. 

### 2.3. Biological Significance

**Figure 3:**
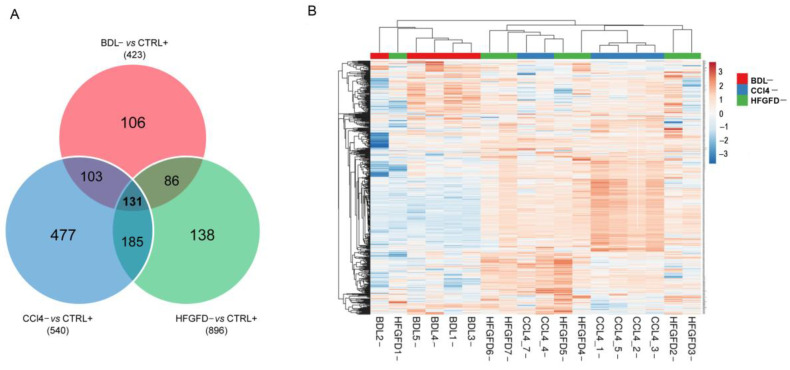
Similarities and differences between dysfunctional LSEC in liver disease models. (**A**) Venn diagram of significantly differentially expressed proteins in CD32b^−^ LSEC from the three liver diseases models compared with CD32^+^ LSEC from control animals. (**B**) Expression profile heatmap of those common differentially expressed proteins in at least two models. BDL, bile duct ligation model; HFGFD, high fat glucose fructose diet model; CCl_4_, carbon tetrachloride model; CTRL, control.

Since we considered the loss of specific marker CD32b as the hallmark of capillarization, we performed another round of analysis comparing the protein expression of CD32b^−^ LSEC from the three models with that of CD32b^+^ LSEC from healthy animals, that is, BDL^−^ vs. CTRL-SD^+^, HFGFD^−^ vs. CTRL-SD^+^ and CCl_4_^−^ vs. CTRL-W^+^, aiming at extracting the information from the two extreme situations. We obtained 426, 540 and 896 differentially expressed proteins in BDL, HFGFD and CCl_4_ models, respectively. Looking for similarities in the capillarization process, differential expression of 131 common proteins was found in CD32b^−^ LSEC from the three models compared to positive controls, while the expression of 505 proteins was shared by at least two models (Figure 3A). On the other hand, to illustrate the differences between models when comparing the expression of these 505 proteins, hierarchical clustering analyses were conducted, with two clear clusters separating the BDL model on one side and CCl_4_ model on the other (Figure 3B). The HFGFD model fell mostly interspersed with CCl_4_, but one sample was also clustered with BDL, suggesting that the HFGFD model has mixed features from both CCl_4_ and BDL.

#### 2.3.1. Enrichment Analysis 

**Figure 4:**
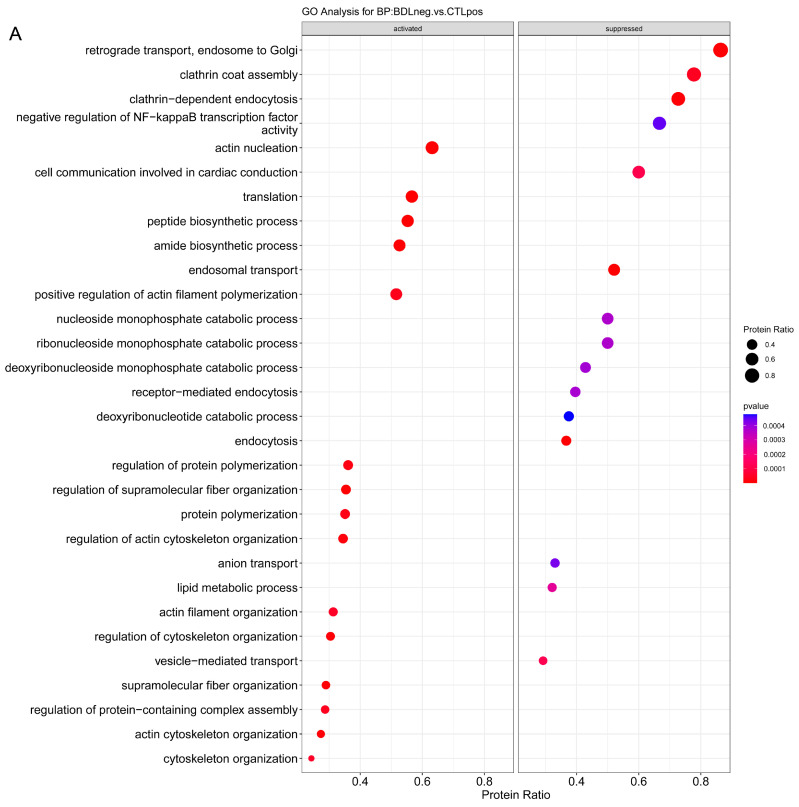

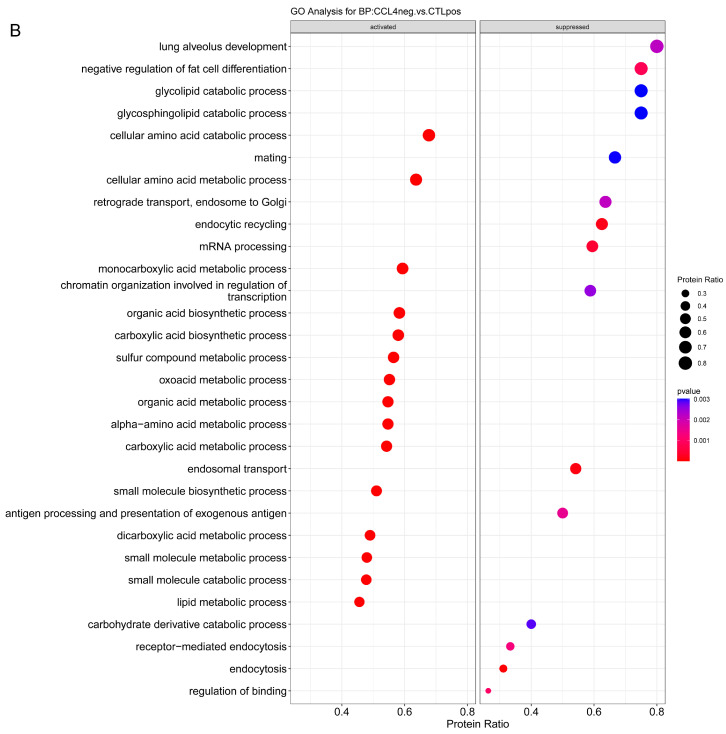

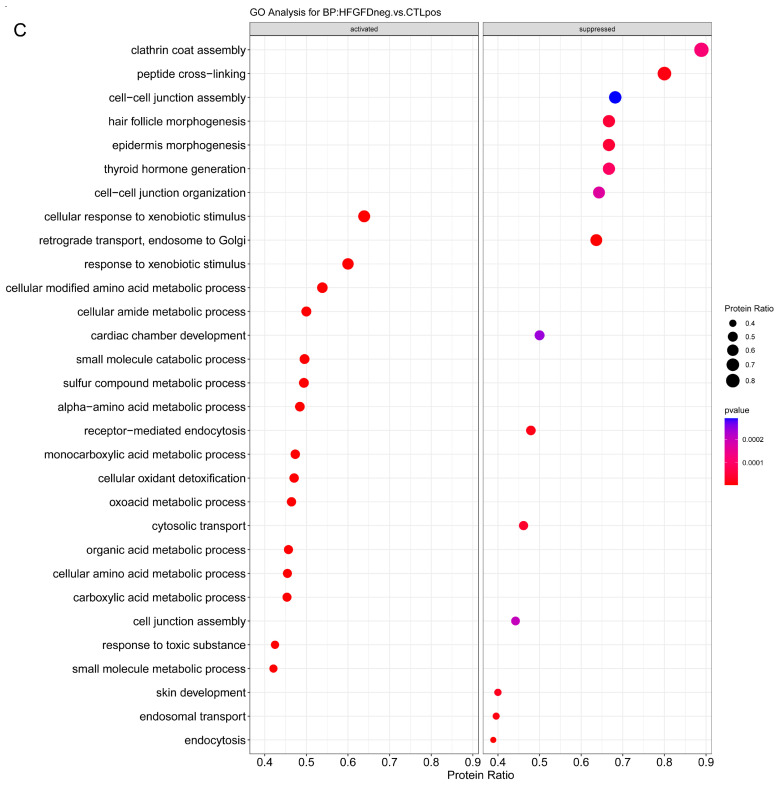
Analysis of biological significance of specific changes in dysfunctional LSEC generated in the three models. Enrichment analysis produced dot plots of the top 15 GO-BP terms of activated and suppressed pathways based on significantly differentially expressed proteins in CD32b^−^ LSEC from (**A**) BDL, (**B**) HFGFD and (**C**) CCl_4_ models compared with CD32b^+^ LSEC from their respective controls. The size of the dots relates the protein ratio, which is the ratio between the proteins in the data that belong to that term and the total number of proteins in the term. The color of the plot refers to significance level. BDL, bile duct ligation model; HFGFD, high fat glucose fructose diet model; CCl_4_, carbon tetrachloride model; CTRL, control.

The analysis of biological significance of protein expression of the CD32b^−^ LSEC from the three models compared with that of their respective CD32b^+^ LSEC controls was based on GSEA over the annotation database Go-BP. Figure 4 shows dot plots of the top 15 GO-BP terms with a positive or negative score found for each model/comparison. Enrichment maps of the top 60 (when available) GO-BP terms found with an adjusted *p*-value ≤ 0.15 for each comparison are shown in Supplementary Figure S1. These maps group the terms by similarity, making it easier for interpretation. A network plot of the proteins found in the top 5 GO-BP terms for each comparison are shown in Supplementary Figure S2A–C. This plot allows visualization of the linkages between proteins and terms since a protein may belong to multiple terms. 

According to GSEA analysis, the model representing a most advanced stage of disease, the BDL model, is defined by the activation of different pathways related to actin cytoskeleton organization, including the synthesis and polymerization of actin filaments; and a consistent suppression of pathways related to clathrin-dependent endocytosis and all the processes related to the endosomal and vesicle transport from the cell membrane to the Golgi apparatus. In addition, there is a down-representation of proteins participating in pathways related to the nucleic acid catabolism (Figure 4A, Supplementary Figures S1A and S2A). In the case of the HFGFD model, representing the first stages of NAFLD-NASH without fibrosis, the enriched activated pathways are related to metabolic processes, mostly of fatty acids and amino acids, but also to the cellular response to xenobiotic processes and toxic substances and to oxidant detoxification. Regarding the suppressed pathways in this model, there is a down-representation of proteins involved in cell–cell junction assembly and organization, and in clathrin- and receptor-mediated endocytosis and cytosolic transport of endosomes to the Golgi (Figure 4B, Supplementary Figures S1B and S2B). Finally, in the CCl_4_ model, representing a non-decompensated stage of cirrhosis, the activated overrepresented pathways are related to metabolic processes, mostly of fatty acid and amino acids, but also of small molecules and sulfur compounds. Likewise, the CCl_4_ model is defined by the suppression of proteins involved in the catabolism of glycolipids and carbohydrates and, once again, in endocytosis and both endocytic recycling and retrograde transport to the Golgi (Figure 4C, Supplementary Figures S1C and S2C).

#### 2.3.2. Overrepresentation Analysis 

An ORA was performed over the GO Pathway database to find the terms enriched in the lists of 131 common differentially expressed proteins between the comparisons obtained in the three liver diseases models, distinguishing up-regulated from down-regulated ones.

**Figure 5:**
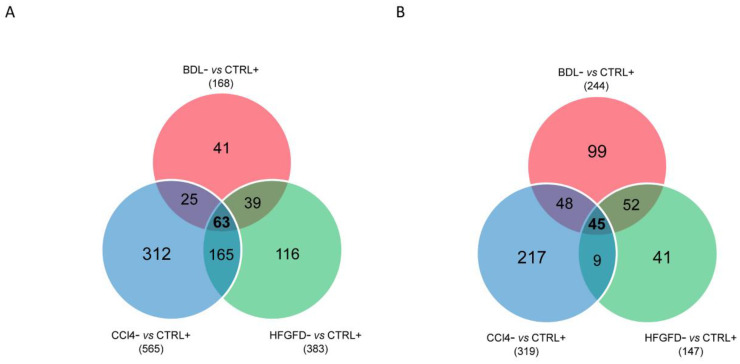

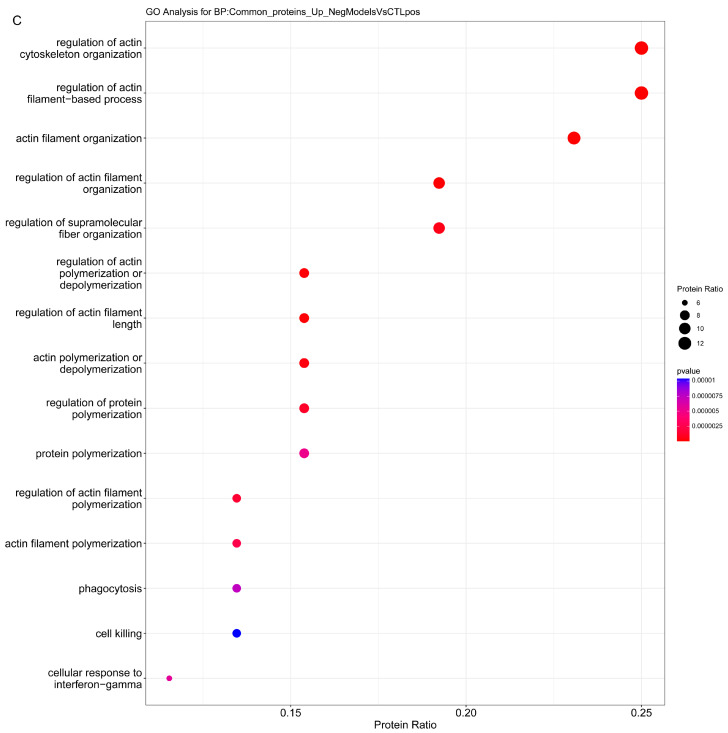

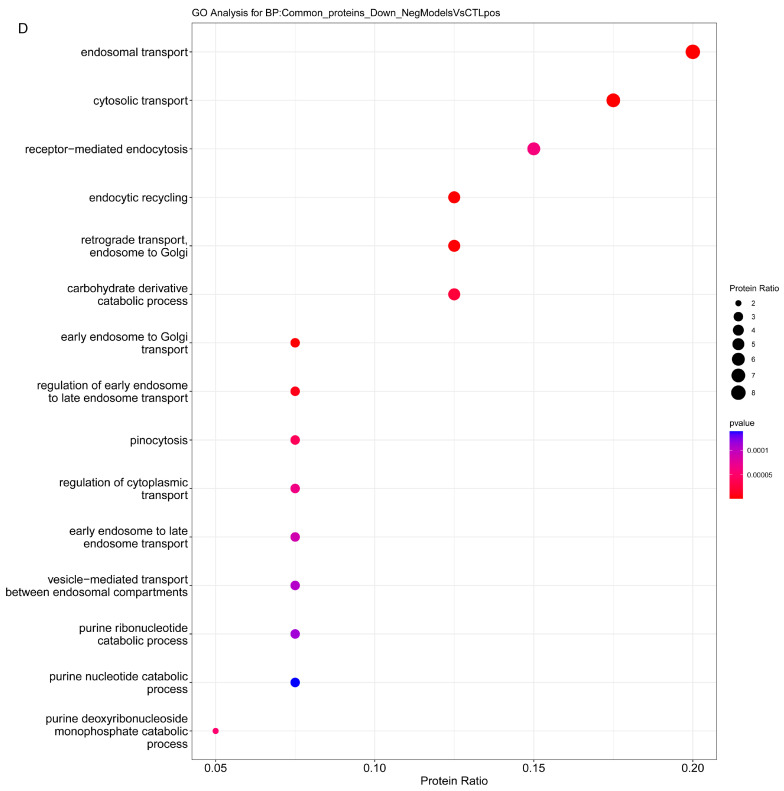
Analysis of biological significance of common changes in dysfunctional LSEC. Overrepresentation analysis performed over GO-BP Pathways databases to find the terms enriched in the lists of differentially expressed proteins in common between the three comparisons analyzed. (**A**,**B**) Venn diagrams of significantly differentially expressed (**A**) up-regulated or (**B**) down-regulated proteins in dysfunctional LSEC from the three models. (**C**,**D**) Dot plots of top 15 GO-BP terms enriched in the common (**C**) 63 up-regulated or (**D**) 45 down-regulated proteins. The size of the dots relates the protein ratio, which is the ratio between the proteins in the data that belong to that term and the total number of proteins in the term. The color of the plot refers to significance level. BDL, bile duct ligation model; HFGFD, high fat glucose fructose diet model; CCl_4_, carbon tetrachloride model; CTRL, control.

A total of 63 proteins were found up-regulated in common between the three models analyzed with a *p*-value ≤ 0.05 and ratio ≥ 1.5, whereas 45 proteins were found down-regulated in common with a *p*-value ≤ 0.05 and ratio ≤ 0.67, as shown in the Venn diagrams in Figure 5A,B. The enrichment analysis was performed for each list separately (up or down-regulated proteins in common). Dot plots of the top 15 GO-BP terms pathways for each comparison are presented in Figure 5C,D. Supplementary Figure S3A,B show the enrichment maps of the top 60 (when available) GO-BP terms found enriched. A network plot of the proteins found in the top five GO-BP terms found for each comparison is in Supplementary Figure S4A,B. The ORA analysis demonstrates clearly that the up-regulated proteins shared by the three models are implicated in pathways related to the actin cytoskeleton organization but also to the role of LSEC as immunomodulatory cells (Figure 5C, Supplementary Figures S3A and S4A). Regarding the common down-regulated proteins between the three models, the ORA analysis shows, as expected, that they are related to all the different steps of the endocytic machinery (Figure 5D, Supplementary Figures S3B and S4B).

A complete list of proteins in top 15 GO-BP terms involved in up-regulation of actin cytoskeleton organization and immune regulation pathways and down-regulation of endocytosis and metabolic/catabolic pathways from the ORA analysis are depicted in Table 2.

**Figure 6:**
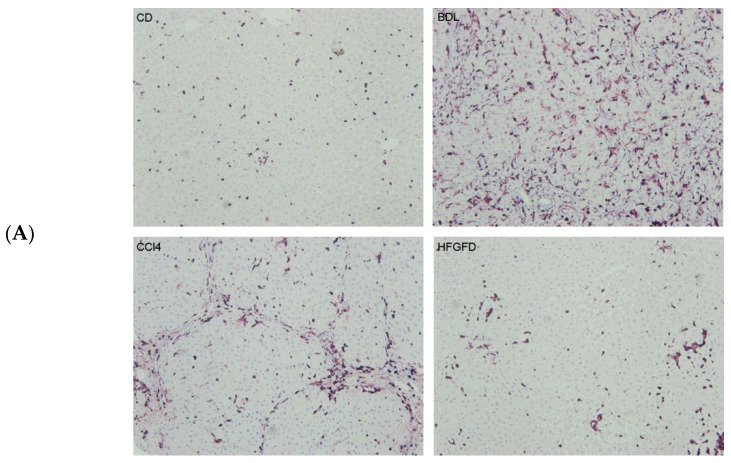

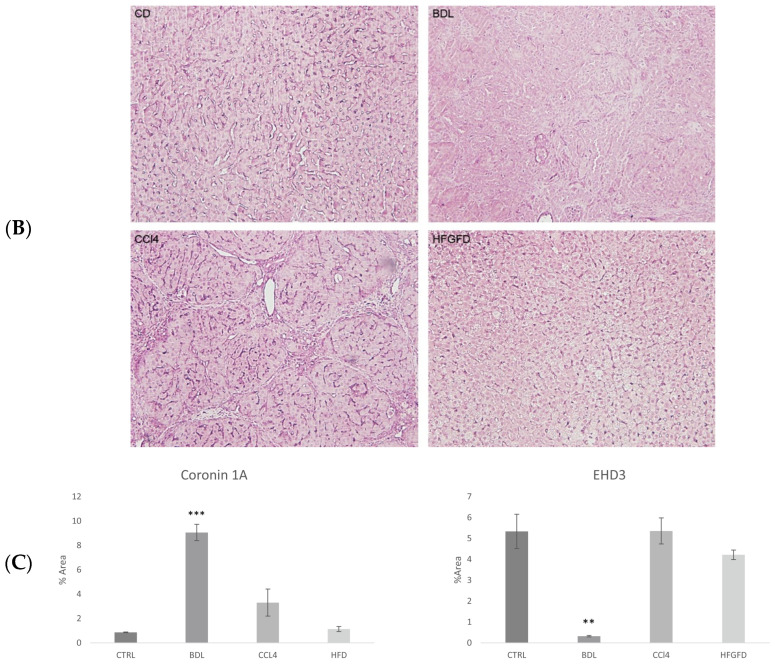
Immunohistochemical assessment of protein expression. Representative images of Coro1a (**A**) and EHD3 (**B**) immunostaining at 10× magnification in liver sections of healthy controls compared to the three rat models of chronic liver disease. Positive staining clearly defines the liver sinusoids. CTRL, control; BDL, bile duct ligation model; CCl_4_, carbon tetrachloride model; HFGFD, high fat glucose fructose diet model; Coro1A, Coronin 1A; EHD3, EH Domain-Containing Protein 3. (**C**) Bar charts showing immunohistochemical quantitation of Coro1a and EHD3 expression levels, represented as mean ± SEM (*n* = 3 per group). ** *p* ≤ 0.01, *** *p* ≤ 0.001 vs. control.

Finally, in order to verify some of these results, immunohistochemistry staining of the top common up-regulated protein Coronin 1A (Coro1a), and the top common down-regulated EH Domain-Containing Protein 3 (EHD3), have been performed in whole liver samples from the three rat models carried out in previous studies and compared with healthy controls. As expected, BDL animals show the highest increase in Coro1a and the lowest decrease in EHD3 (Figure 6), in accordance with the near 50% number of CD32b^−^ LSEC isolated in this model (Table 1). 

## 3. Discussion

In this study, we have conducted an exhaustive analysis of differential protein expression of dysfunctional LSEC in the most common experimental models of liver disease (i.e., BDL, HFGFD and CCl4), trying to define molecular markers of dedifferentiation that are particular to certain pathophysiological processes. The results show that, although substantial differences in dysfunctional LSEC from the three models are evident, there are also central similarities that might be fundamental to the progression of liver injury.

Finding biomarkers that reliably distinguish healthy from damaged LSEC in acute or chronic liver injury might add relevant information to a largely unexplored area with few relevant developments thus far [16]. For example, diminished or no expression of CD32b receptors has been largely documented as a hallmark of capillarization [17], and it has been suggested that therapies specifically aimed at reverting this dysfunctionality could help to slow down the entire pathological process [18]. Notwithstanding this, to the best of our knowledge, our group is the first that has conducted studies to elucidate the pathophysiological and phenotypic implications of CD32b^−^ LSEC in various models and pathological scenarios. 

For instance, in a preclinical dietary model in rats reproducing the key phenotypic features of early stages of NASH [19], we showed a significant increase in the percentage of CD32b^−^ LSEC individualized by cellular sorting [20]. We also demonstrated that treatment with statins has an important effect only on de-differentiated CD32b^−^ LSEC, causing phenotype restoration and inducing an improvement in intrahepatic resistance and portal pressure, histological reversal of NASH and inactivation of hepatic stellate cells. In contrast, we did not find any effect of statins on CD32^+^ LSEC [20].

The first remarkable finding of the present work is that the percentage of dysfunctional LSEC was notably different across the three models studied, BDL representing the more advanced/decompensated disease stage—the one with a more pronounced increase of this cellular type. Recent publications described how heterogeneous hepatic endothelial cell populations, comprising two distinct subsets of LSEC, are arranged following the acinar pattern, located differentially to fulfill distinct physiological functions under normal conditions: pericentral zone LSEC, with great expression of CD32b; and periportal zone LSEC, with little or no expression of CD32b [21,22]. Since the CCl4 model showed a low number of CD32b^−^ LSEC, non-significantly different from the number of CD32b^−^ LSEC present in its control, we wondered whether the CD32b^−^ LSEC in the CCl4 model were the same subset of periportal LSEC with low expression in CD32b present in healthy controls or rather the product of a de-differentiation process during hepatotoxic induction. Our results showed that LSEC from the CCl4 model had the highest number of differentially expressed proteins, which largely differed from those of healthy CD32b^−^ LSEC, thus strongly pointing to the second option. Overall, our findings suggest that the capillarization process might display different features depending on the etiology, disease stage and mechanisms causing liver injury. 

The results of the present study add to the evidence suggesting that characterization of LSEC de-differentiation state through protein expression profiles might be used as a proxy of the capillarization process. The comparative analysis carried out between each LSEC subtype (CD32b^+^ and CD32b^−^) with their respective counterparts in the healthy controls from the three models demonstrated that CD32b^−^ LSEC samples display a higher number of differentially expressed proteins than CD32b^+^ LSEC, with the latter being more similar to their control CD32b^+^ LSEC samples. However, CD32b^+^ populations from the three models are indeed also different from their controls and likely represent an intermediate state of de-differentiation towards a more capillarized state. 

Differential proteomic expression also led to an interesting observation, namely that LSEC from models displaying less advanced liver injury (i.e., HFGFD and CCl4) showed similar protein expression that differentiated them from a more advanced CLD (i.e., decompensated cirrhosis) yielded by BDL. Moreover, we demonstrated that the changes in the proteomic profile due to modeling are far more important than the bias due to the background. Although comparison of protein expression was always performed considering the different backgrounds (BDL and HFGFD vs. CTRL-SD and CCl4 vs CTRL-W), HFGFD- and CCl4-sorted LSEC clustered together separately from the BDL model, even though HFGFD and BDL share the same background different from that of CCl4. However, it could also be possible that the distinct protein expression profile is not exclusively due to the advanced stage of CLD but to the characteristics of the mechanisms of liver injury. BDL is an aggressive, short model that induces decompensated cirrhosis in just 4 weeks, with an acute onset of bile accumulation in the liver that might also be at work in this model. In short, the more prominently affected pathways by the differential protein expression in LSEC we observed in the BDL model involved a suppression of LSEC scavenging properties. This is consistent with the main mechanisms of liver injury at play in the BDL model, i.e., LSEC dedifferentiation and angiogenesis, activation of HSCs and rapidly progressing fibrosis, which result in hepatic hemodynamic derangements leading to decompensation (mostly ascites) [7,23]. Yet, it remains to be elucidated whether changes in LSEC protein expression profiles also reflect specific pathways related to obstructive cholestasis. 

As for the biological significance of the pathways related to differential protein expression profile in each model, the most common finding concerned pathways involved in the suppression of the whole endocytic machinery and endothelial dysfunction leading to liver fibrosis. LSEC, having lost the CD32b marker, presented 45 down-regulated proteins, mainly belonging to top protein pathways encompassing receptor-mediated endocytosis and endosomal transport either to the trans-Golgi network or back to the cell surface [24,25]. As we sorted dysfunctional LSEC by CD32b negativity, we expected to obtain cells with suppressed clathrin-mediated endocytosis. We observed that the loss of this receptor was associated with the suppression of proteins involved in the intracellular vesicle transport apparatus for removal of large molecules and nanoparticles from the blood and the metabolic/catabolic processes of these scavenged molecules [7,26]. Likewise, the 63 common up-regulated proteins were related to the activation of pathways associated with the actin-dependent cytoskeleton reorganization regulated by Rho and controlling LSEC fenestration [27,28]. LSEC, having lost the CD32b receptor, showed a potent activation of proteins that modulate the cytoskeleton and the polymerization/depolymerization of actin filaments associated with hepatic stellate cell activation and proteins regulating the immune response. Alterations in the number or diameter of fenestrae have important implications for hepatic function in liver diseases. It has been recently demonstrated [29] that the cytoskeleton is closely associated with fenestrae and that large stress actin fibers, which are formed during LSEC dedifferentiation, are organized in dense rings surrounding fenestrae. More importantly, in vitro dedifferentiated LSECs that have lost fenestrae are able to re-form such actin fibers. Fenestrae loss is an early event in liver cirrhosis and is common to all the three models representing different etiologies. Control of the fenestrae aperture could be an important element to help reverse the fibrosis process. In this sense, an analysis of LSEC porosity through calculating fenestrae size and frequency by means of transient electron microscopy [30] would be an elegant proof of how these models differ from each other regarding LSEC morphology.

There are two important limitations to our study. First, the choice of CD32b loss as a hallmark of dysfunctional LSEC is based on previous works describing LSEC in health and disease [11,31]. However, the chronology of events in different etiologies and the molecular mechanisms driving dedifferentiation have not been fully elucidated. The fact that BDL and CCl4 models significantly differ in the number of CD32b^−^ LSEC suggests that dysfunctional LSEC could still be CD32b^+^ and that other molecular markers might be considered depending on the etiology.

Another aspect to consider is that this study has been carried out in animal models of CLD and there are no data of comparison with human samples. In view of that, we consider that isolating and sorting enough LSEC from human biopsies to perform a proteomic study is nowadays virtually impossible. 

In summary, substantial differences but also similarities in dysfunctional LSEC from the three most common models of CLD have been described, supporting the idea that in different etiologies/disease stages, LSEC may harbor different protein expression profiles. Our findings provide a preliminary rationale to further investigate protein profiles of dysfunctional LSEC as biomarkers of liver disease. Future studies should include in-depth analyses of deregulated proteins and pathways in each experimental liver disease model in order to consolidate the understanding of the role of particular LSEC phenotypes while allowing to identify specific therapeutic targets that boost further drug development. 

## 4. Material and Methods

### 4.1. Experimental Models of Liver Disease 

Cirrhosis was induced by bile duct ligation (BDL) to mimic an advanced decompensated CLD. Male Sprague-Dawley rats (Charles River Laboratories, L’Arbresle, France) weighing 200–220 g were anesthetized with inhaled isoflurane, and the common bile duct was occluded by double ligature with a 4–0 silk thread. The bile duct was then resected between the two ligatures. Animals received weekly intramuscular vitamin k1 to decrease mortality from bleeding [32].

A diet-induced rat model reproducing the key phenotypic features of non-alcoholic steatohepatitis (NASH) early stages (obesity, insulin resistance, intestinal dysbiosis, endothelial dysfunction and portal hypertension) was also performed [19]. Male Sprague-Dawley rats weighing 230–260 g were fed ad libitum with a high fat glucose–fructose diet (HFGFD) for 8 weeks. HFGFD consisted of 30% fat (butter, coconut oil, palm oil, beef tallow) with mainly saturated fatty acids (5.73 Kcal/g), supplemented with cholesterol (1 g/Kg) (Ssniff Spezialdiaten GmbH, Soest, Germany), and a beverage of glucose–fructose (42 g/L, 45% glucose-55% fructose). 

Male Sprague-Dawley rats were the healthy controls (CTRL-SD) for both BDL and NASH models, following a control diet (CD) for 8 weeks. CD consisted of a grain-based chow with 4% fat (2.89 Kcal/g) (Teklad 2014, Harlan laboratories, Indianapolis, IN, USA) and tap water. 

To obtain an early, non-decompensated model of cirrhosis, male Wistar rats (Charles River Laboratories, L’Arbresle, France) weighing 100–120 g followed a CCl4 inhalation protocol [33] for 14 weeks. Phenobarbital (0.3 g/L) was added to drinking water one week before the inhalation protocol. Male control Wistar rats (CTRL-W) also received phenobarbital in drinking water but did not follow the CCl4 inhalation protocol.

The animals were kept in environmentally controlled animal facilities at the Cellex Center (Barcelona, Spain), housed under 12 h light/dark cycles at constant temperature and humidity. All procedures were conducted in accordance with European Union Guidelines for Ethical Care of Experimental Animals (EC Directive 86/609/EEC for animal experiments) and approved (file numbers: 11244, 11014 and 10989) by the Animal Care Committee of the Vall d’Hebron Institut de Recerca.

### 4.2. LSEC Isolation 

LSEC were isolated from livers of CTRL-SD (*n* = 6), CTRL-W (*n* = 5), BDL (*n* = 7), HFGFD (*n* = 7) and CCl4 (*n* = 7) rats as previously described [20]. Briefly, livers were perfused with collagenase, excised and digested. Resulting cells were filtered and centrifuged to eliminate hepatocytes. Then, the supernatant was centrifuged in a two-phase Percoll gradient (25%/50%). The central fraction containing LSEC and Kupffer cells was collected and seeded in a non-coated plate for 30 min. Non-adherent LSEC were seeded in collagen-coated culture plates, incubated for 45 min and washed afterwards (see Supplementary Material for further details).

### 4.3. Fluorescence-Activated Cell Sorting 

LSEC were harvested, trypsinized and washed with PBS 5% FBS. Of all LSEC obtained, 105 cells were used for each of the control conditions and the remaining cells were processed for double labeling, incubated with fluorescent labeled antibodies against CD32b·FITC (1:10; Novus Biologicals Littleton, CO, USA) and CD11b/c·APC (1:10; Miltenyi Biotec, Bergisch Gladbach, Germany) for 1 h and resuspended in 500 μL of PBS 5% FBS with DAPI (2 µL/106 cells). Two-color flow cytometry was performed on a FACS-Aria flow cytometer (BD Biosciences, San Jose, CA, USA). Voltages were based on unstained cells, and compensation was set using single-stained positive controls for each color. Viable cells were sorted by DAPI staining, doublets and aggregates were excluded from analysis based on the forward scatter (FSC)-H and FSC-A profile, and KC or macrophages were excluded by gating CD11b/c cells. The rest of the cells, either CD32b^+^ or CD32b^−^, were sorted, collected and washed with PBS (to wash off FBS) for the proteomic study, and results were evaluated with FCS Express 4 Flow Research Edition. 

### 4.4. Differential Proteomic Study

The samples of sorted CD32b^+^ and CD32b^−^ LSEC were shipped in dry ice to the Proteomics platform of CIC-BioGUNE (Derio, Spain) to obtain their full proteomic profile by performing a label-free relative protein quantification through nLC MS/MS. Cells were lysed in a buffer containing 7M Urea 2M Thiourea and 4% CHAPS, and the protein obtained was digested with trypsin using the SP3 method (single-pot solid-phase-enhanced sample preparation). The resulting peptides were loaded onto an Evosep One chromatograph (30 SPD protocol) coupled on-line to a timsTOF Pro mass spectrometer (Bruker) that uses parallel accumulation serial fragmentation (PASEF) acquisition to provide extremely high speed and sensitivity. The data obtained were then processed with PEAKS software, X PRO version (Biofinformatics Solutions Inc, Waterloo, Canada); this software identifies proteins and performs an intensity-based quantification of proteins. Proteins identified with at least two different peptides (one of them being unique) at a False Discovery rate (FDR) < 1% were considered for further analyses. Proteins were regarded as differentially abundant with a ratio ≥ 1.5 (overexpressed) or ≤0.67 (under-expressed). Then, further refinement was accomplished by filtering through the *p*-value from a paired Student’s *t*-test < 0.05. Perseus software 1.5.1.5 (free from Max Plank Institute, Munich) was used for the differential protein abundance analyses. Principal Component Analysis (PCA) and heatmaps for data visualization were performed using Clustvis software v1.0 (http://biit.cs.ut.ee/clustvis/, accessed between 10 October and 19 November 2021. Further details are available in the Supplementary Material. 

### 4.5. Immunohistochemistry

Immunohistochemistry detection of Coro1a and EHD3 was carried out on 4 µm sections of rat livers from three chronic liver diseases models and controls. Nonspecific antibody binding was prevented by incubation with 1:20 dilution of goat serum. Sections were then incubated with primary antibodies against Coro1a at 1/1000 dilution (ab203698, Cambridge, UK) and EHD3 at 1/300 dilution (25320-1-AP, proteintech, Manchester, UK), followed by EnVision + Dual Link System-HRP (Dako, Glostrup, Denmark), and visualized with the VIP substrate kit (Vector, Burlingame, CA, USA) that produces a purple precipitate. The samples were counterstained with hematoxylin. Quantitative analysis of stained areas was carried out with Image J software41 on ten fields per sample, randomly captured at 10× magnification with an optical microscope Olympus BX61 (Olympus, Hamburg, Germany).

### 4.6. Analysis of the Biological Significance

To analyze the biological significance of differential protein expression in LSEC subtypes from the different rat models of liver damage performed in this study, we compared the extreme situations, that is, the expressed proteins from CD32b^−^ LSEC from each model versus those obtained from CD32b^+^ LSEC from their respective controls (BDL.CD32b^−^ vs. CTRL-SD.CD32b^+^; HFGFD.CD32b^−^ vs. CTRL-SD.CD32b^+^; CCL_4_.CD32b^−^ vs. CTRL-W.CD32b^+^). The lists of differentially expressed proteins obtained in each case were used to generate Top Tables containing the *p* values and ratio statistics for each comparison. Then, a Gene Set Enrichment Analysis (GSEA) was performed to determine the pathways in which these proteins were involved, based on Gene Ontology (Biological Process category, GO-BP), using direct protein annotations. This analysis determines whether the presence of functionally related proteins is explained by chance alone, or whether there is an enrichment of proteins related to that particular function among the proteins that have been shown to change significantly in our comparison with respect to a reference set (all the proteins analyzed in the study) [34]. Different lists for each model were generated, sorted by *p*-values and filtered by an adjusted *p*-value ≤ 0.15. A positive enrichment score indicates that the term enriched is mainly composed of up-regulated proteins, whereas a negative score indicates the opposite.

Finally, an overrepresentation analysis (ORA) was also performed over GO-BP terms databases with the lists of up-/down-regulated proteins found in common between the three models analyzed. Like GSEA analysis, the results of the ORA enrichment analysis produce lists sorted by enrichment *p*-value and filtered by an adjusted *p*-value ≤ 0.15. 

### 4.7. Biostatistical Analysis

Cell sorting and differential proteomic study results were analyzed using Excel, R and GraphPad Prism 8 (GraphPad Software, Inc., La Jolla, CA, USA).

Quantitative results were expressed as mean ± standard error of the mean (SEM). Statistical analyses were performed with unpaired Student’s *t*-test (between two groups) or one-way analysis of variance (ANOVA) with Bonferroni’s post-hoc correction (among three or more groups), using GraphPad Prism 8. A value of *p* < 0.05 was considered statistically significant.

The statistical analysis for the biological significance was performed using the statistical language “R” (R version 4.1.1 (10 August 2021), Copyright (C) 2018 The R Foundation for Statistical Computing) and libraries from the Bioconductor Project (www.bioconductor.org, accessed between 11 and 17 June 2022). Specifically, enrichment analyses were performed with clusterProfiler R package v4.0.5 [35].

## Figures and Tables

**Table 1:** Number of animals and sorted LSEC used in this study.

Model/Strain	N	N of Isolated LSEC	LSEC CD32b^+^ (%)	LSEC CD32b^−^ (%)
CTRL/SD	6	1,235,600 ± 188,804	95.5 ± 1.3	4.5 ± 1.3
CTRL/W	5	1,074,100 ± 240,424	94.3 ± 0.6	5.7 ± 0.6
BDL/SD	7	319,000 ± 124,439	55.4 ± 8.7	44.6 ± 8.7 ****
HFGFD/SD	7	1,774,100 ± 373,974	83.1 ± 4.5	16.9 ± 4.5
CCl_4_/W	7	3,319,375 ± 585,295	94.0 ± 0.7	6.0 ± 0.7

**** *p* < 0.0001 compared to CTRL/SD. BDL, bile duct ligation model; HFGFD, high fat glucose fructose diet model; CCl_4_, carbon tetrachloride model; CTRL, control; SD, Sprague-Dawley; W, Wistar.

**Table 2:** List of common up and down-regulated proteins in CD32b^−^ LSEC in the three models.

Up-Regulated			
Pathway	Accession #	Symbol	Protein Name
Actin cytoskeleton organization	Q91ZN1	Coro 1a	Coronin-1A
	B2GV73	Arpc3	Actin-related protein 2/3 complex subunit 3
	P35465	Pak1	Serine/threonine-protein kinase PAK 1
	Q5RKI0	Wdr1	WD repeat-containing protein 1
	Q6AYC4	Capg	Actin regulatory protein CAP-G
	O08719	Evl	Ena/vasodilator-stimulated phosphoprotein-like
	G3V7Q7	Iqgap1	IQ motif-containing GTPase-activating protein 1
	Q68FP1	Gsn	Gelsolin
	B0BMY7	Twf2	Twinfilin actin-binding protein 2
	Q5M860	Arhgdib	Rho GDP dissociation inhibitor beta
	Q99N37	Arhgap17	Rho GTPase-activating protein 17
	Q64303	Pak2	Serine/threonine-protein kinase PAK 2
	Q68FX4	Hcls1	Hematopoietic cell specific Lyn substrate 1
	P48675	Des	Desmin
	P31000	Vim	Vimentin
	Q5XI38	Lcp1	Lymphocyte cytosolic protein 1
Immune regulation	Q5RKI0	Wdr1	WD repeat-containing protein 1
	P04157	Ptprc	Receptor-type tyrosine-protein phosphatase C, CD45
	P81718	Ptpn6	Tyrosine-protein phosphatase non-receptor type 6
	P01026	C3	Complement C3
	G3V726	Gzmm	Granzyme M
	Q91ZN1	Coro1a	Coronin-1A
	Q64725	Syk	Tyrosine-protein kinase SYK
	P32577	Csk	Tyrosine-protein kinase CSK
	P04041	Gpx1	Glutathione peroxidase 1
	Q5XI38	Lcp1	Lymphocyte cytosolic protein 1
	P04797	Gapdh	Glyceraldehyde-3-phosphate dehydrogenase
**Down-Regulated**			
**Pathway**	**Accession #**	**Symbol**	**Protein Name**
Endocytosis	Q8R491	Edh3	EH domain-containing protein 3
	Q641Z6	Edh1	EH domain-containing protein 1
	Q8R3Z7	Edh4	EH domain-containing protein 4
	B1H267	Snx5	Sorting nexin-5
	A3RLA8	Fcgr2b	Fc gamma receptor 2B, CD32b
	Q5U211	Snx3	Sorting nexin-3
	B2RYP4	Snx2	Sorting nexin-2
	Q99N27	Snx1	Sorting nexin-1
	O88797	Dab2	Disabled homolog 2
	Q6AYE2	Sh3glb1	Endophilin-B1
	P62744	Ap2s1	AP-2 complex subunit sigma
	B0BNK1	Rab5	Member RAS oncogene family
	P07154	Ctsl	Procathepsin L
	P63045	Vamp2	Vesicle-associated membrane protein 2
Metabolic- Catabolic	Q920P6	Ada	Adenosine deaminase
	Q9JKB7	Gda	Guanine deaminase
	Q8R491	Ehd3	EH domain-containing protein 3
	O88797	Dab2	Disabled homolog 2
	Q6AYE2	Sh3glb1	Endophilin-B1
	Q99N27	Snx1	Sorting nexin-1
	Q6IRK9	Cpq	Carboxypeptidase Q Liver annexin-like protein 1
	P07154	Ctsl	Procathepsin L
	D3ZF77	Akr1c15	Aldo-keto reductase family 1 member C15
	F1LR10	Lima1	LIM domain and actin-binding protein 1
	Q6IMY6	Lipa	Lipase
	P50442	Gatm	Glycine amidinotransferase
	Q01062	Pde2a	cGMP-dependent 3′,5′-cyclic phosphodiesterase
	Q9EPB1	Dpp7, Dpp2	Dipeptidyl peptidase 2 Dipeptidyl peptidase 7
	Q9EQV6	Tpp1, Cln2	Tripeptidyl-peptidase
	Q6AXR4	Hexb	Beta-hexosaminidase subunit beta

## Data Availability

The mass spectrometry proteomics data have been deposited into the ProteomeXchange Consortium via the PRIDE partner repository (https://www.ebi.ac.uk/pride) under the dataset identifier PXD040661.

## Supplementary Materials

The following supporting information can be downloaded at: https://www.mdpi.com/article/10.3390/ijms241511904/s1.
