# Supplementary material for: Proteomic Analysis of Dysfunctional Liver Sinusoidal Endothelial Cells Reveals Substantial Differences in Most Common Experimental Models of Chronic Liver Diseases
Source: Int J Mol Sci. 2023 Jul 25;24(15):11904. doi: 10.3390/ijms241511904 (PMC10418749; doi:10.3390/ijms241511904)
Supplement: Supplementary file 1 [file ijms-24-11904-s001.zip › SUPPLEMENTARY DATA.pdf]

SUPPLEMENTARY DATA AND LEGENDS TO SUPPLEMENTARY FIGURES

LSEC isolation

LSEC were isolated from rat livers of three animal models (BDL, HFGFD and CCl4), as well as from healthy rat livers of both controls (SD and W). Briefly, after rats were anesthetized with ketamine hydrochloride (Ketolar 50 mg/mL, Parke-Davis S.L./Pfizer, Madrid, Spain) and midazolam (Normon S.A., Madrid, Spain), livers were perfused through the portal vein for 10 min at a flow rate of 20 mL/min at 37ºC with Hanks' Balanced Salt Solution (HBSS, Capricorn Scientific GmbH, Ebsdorfergrund, Germany) without calcium and magnesium, containing 12 mM Hepes (pH 7.4), 0.6 mM EGTA and 0.23 mM BSA, divided in 4 centrifuge tubes. Just before starting the perfusion, 0.9 mL 1% heparin were added into the two first centrifuge tubes (0.5 mL 1st tube and 0.4 mL 2nd tube) to prevent blood clotting. Before continuing with the *in vivo* digestion of the liver, a small fraction of the liver was excised for the subsequent histological analyses. Then, 50 mL of HBSS containing 12 mM Hepes (pH 7.4), 4 mM $CaCl_2 \cdot 2H2O$, and 3 mL of collagenase A (10 mg/mL; for cirrhotic livers the volume was increased to 3.9 mL) (Roche Diagnostics, Germany) flowed at a rate of 10 mL/min at 37ºC. Approximately 5 min later, the thread of the inferior hepatic cava vein was clamped and the liver perfused with the remaining buffer (150 mL), at a flow rate of 5 mL/min at 37ºC. The resultant digested liver was excised and mechanically disrupted, and *in vitro* digestion was performed with the same buffer at 37ºC for 10 min in constant agitation. The cells were then passed through 100 µm nylon filters (VWR International, USA). The filtrate was divided in 4 centrifuge tubes containing 25 mL of cold Krebs' buffer (25 mM Hepes (pH 7.4) and 2.5 mM $CaCl_2 \cdot 2H2O$) refrigerated at 4ºC, and the tubes were centrifuged at 50 g for 4 min at 4ºC. Once the centrifugation was finished, the supernatant containing the non-parenchymal cells (LSEC and KC) was stored in 4 new centrifuge tubes while the pellet containing the hepatocytes was discarded. This centrifugation was performed a second time to eliminate the hepatocytes more thoroughly. The supernatant was then centrifuged at 800 g for 10 min, and the obtained pellet was resuspended in Dulbecco's Phosphate Buffered Saline (PBS, Capricorn Scientific GmbH, Ebsdorfergrund, Germany) and centrifuged again at 50 g for 4 min, to completely discard the hepatocytes that were in the pellet. The subsequent supernatant was then centrifuged at 800 g for 10 min, and the obtained pellet was resuspended with PBS to a final volume of 10 mL. The resulting solution was centrifuged at 800 g for 25 min through a two-step Percoll gradient (25–50%) (Sigma-Aldrich, USA) at 4ºC, with a lowered acceleration in half and deceleration without brake. The interphase of the gradient, enriched in LSEC and KC, was obtained, rinsed with PBS and centrifuged at 800 g for 10 min. The cell pellet was resuspended in tempered RPMI 1640 Medium (Biowest, France) (supplemented with 10% FBS, 1% penicillin-streptomycin, 1% L-glutamine, 1% amphotericin B, 1% ECGS, and 1% heparin), seeded in a plastic Petri dish and incubated for 30 min at 37ºC in humid atmosphere with 5% CO_2, in order to separate LSEC from KC by their selective adherence. To isolate LSEC,

after adhesion of KC in the Petri dish, the supernatant containing only endothelial cells was collected. Finally, to minimize cell contamination, LSEC were seeded in a collagen-coated Petri dish (100 µg/mL) and incubated for 45 min (37ºC, 5% CO_2). After this time, LSEC were already adhered to the Petri dish and we could work with the primary culture.

Differential proteomic study

The samples of sorted CD32b+ and CD32b- LSEC were shipped in dry ice to the Proteomics platform of CIC-bioGUNE (Derio, Spain) to obtain their full proteomic profile by performing a label-free relative protein quantification through nLC-MS/MS. Protein was extracted by incubating cells in a buffer containing 7M urea, 2M thiourea, and 4% CHAPS. Samples were incubated in this buffer for 30 min at RT under agitation and digested following the SP3 protocol described by Hughes et al. 2014 with minor modifications. Briefly, this protocol involves the use of carboxylate-coated paramagnetic beads that bind protein and peptides in an unbiased fashion. Protein was bound to the beads in 70% acetonitrile, then washed twice using 70% ethanol. Next, trypsin was added in 50mM ammonium bicarbonate to a trypsin:protein ratio of 1:10, and the mixture was incubated for 2h at 37oC. Both protein and peptides elute from the magnetic beads in ammonium bicarbonate, so digestion and peptide recovery are easy to achieve. After recovery, peptides were dried out in an RVC2 25 speedvac concentrator (Christ) and resuspended in 0.1% FA. Peptides were desalted and resuspended in 0.1% FA using C18 stage tips (Millipore) prior to acquisition.

The resulting peptides were loaded onto an EvoSep One (EvoSep) chromatograph coupled on-line to a TIMS tof Pro mass spectrometer (Bruker), that uses Parallel Accumulation Serial Fragmentation (PASEF) acquisition to provide extremely high speed and sensitivity. 30 SPD protocol (approx. 44min. runs) was used, under default Evosep settings.

The obtained data was then processed with PEAKS software (Biofinformatics Solutions Inc) for protein identification and quantification. Searches were carried out against a database consisting of rat protein entries from Uniprot, and default search parameters were applied (20ppm precursor tolerance, 0.05 Da fragment tolerance, Carbamidomethylation of cysteines as fixed modification, Oxidation of methionines as variable modification). PEAKSQ module was used for the area-based protein quantification. Only proteins identified with at least one peptide at FDR<1% were considered for the analysis.

Quantitative protein data were loaded onto Perseus software (free software from Max Plank Institute, Munich). This program was used for the differential protein abundance analyses. For this purpose, protein abundance data were log2 transformed, filtered based on reproducibility (proteins present in at least 70% of the samples of one of the groups were kept in the analysis) and imputated (missing values were substituted by abundances randomly taken from the 10% least abundant proteins in each sample).

Supplementary Figure S1. Enrichment map of the top 60 (when available) GO-BP terms found enriched with an adjusted p-value ≤ 0.15 for A) BDL, B) HFGFD and C) CCl_4 model comparison lists from the Protein Set Enrichment analysis (GSEA). These maps group the terms by similarity, making it easier for interpretation. Nodes are colored by p-value and their size reflects the number of proteins found in that term.

Supplementary Figure S2. Network plot of the proteins found in the top 5 GO-BP for A) BDL, B) HFGFD and C) CCl_4 model comparison lists from the GSEA analysis. These plots allow visualization of the linkages between proteins and terms since a protein may belong to multiple terms. The size of the term nodes is related to the number of proteins found in that term and the color of the edges links each protein with its term.

Supplementary Figure S3. Enrichment map of the top 60 (when available) GO-BP terms found enriched with an adjusted p-value ≤ 0.15 for A) upregulated and B) downregulated proteins common to the three models from the ORA analysis. These maps group the terms by similarity, making it easier for interpretation. Nodes are colored by p-value and their size reflects the number of proteins found in that term.

Supplementary Figure S4. Network plot of the proteins found in the top 5 GO-BP for A) up-regulated and B) down-regulated proteins common to the three models from the ORA analysis. These plots allow visualization of the linkages between proteins and terms since a protein may belong to multiple terms. The size of the term nodes is related to the number of proteins found in that term and the color of the edges links each protein with its term.
