# Supplementary material for: Proteomic Analysis of Dysfunctional Liver Sinusoidal Endothelial Cells Reveals Substantial Differences in Most Common Experimental Models of Chronic Liver Diseases
Source: Int J Mol Sci. 2023 Jul 25;24(15):11904. doi: 10.3390/ijms241511904 (PMC10418749; doi:10.3390/ijms241511904)

Suplemmentary Figure S1A

(GSEA 60 top BDL)

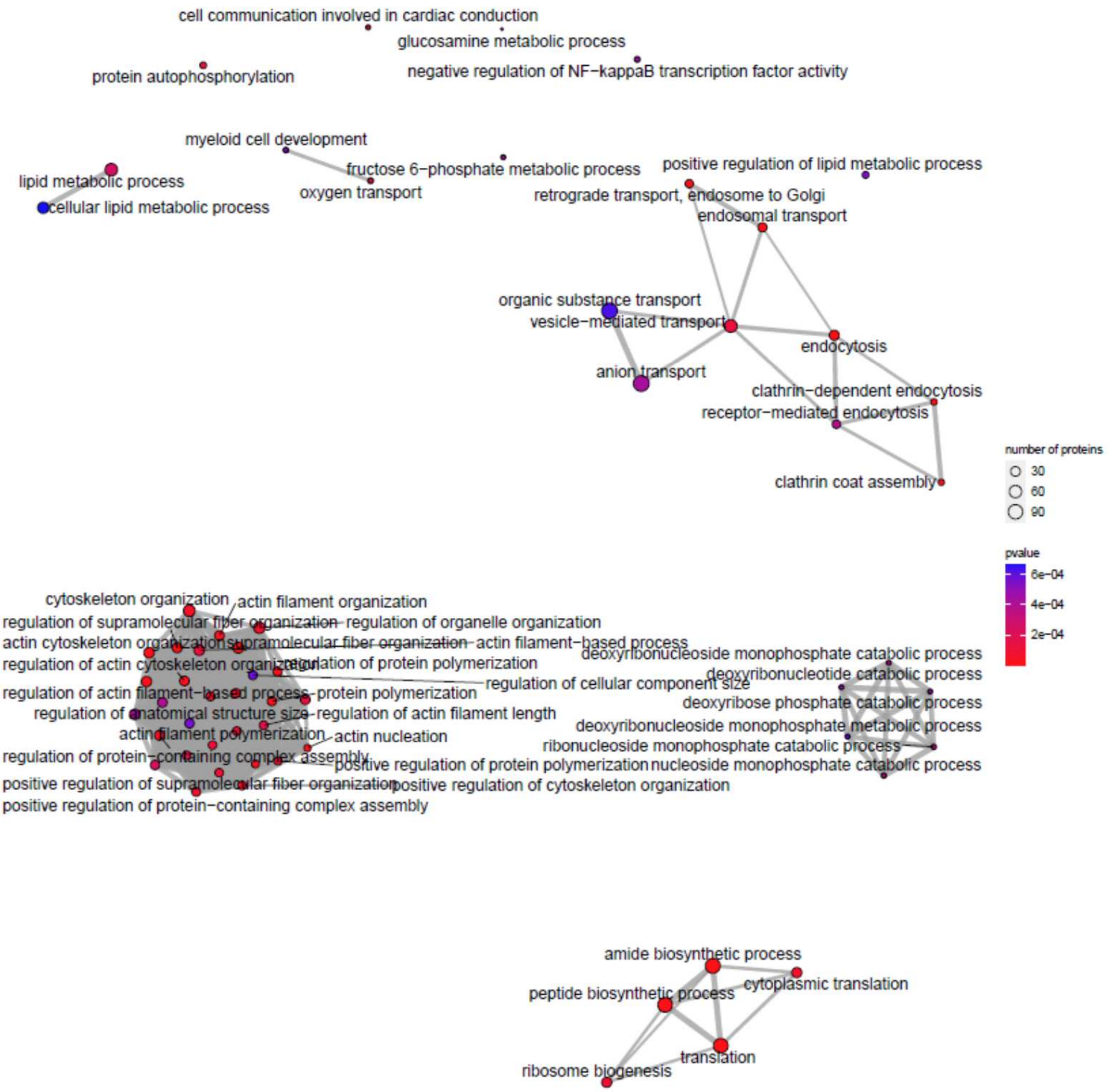

Suplemmentary Figure S1B

(GSEA 60 top HFGFD)

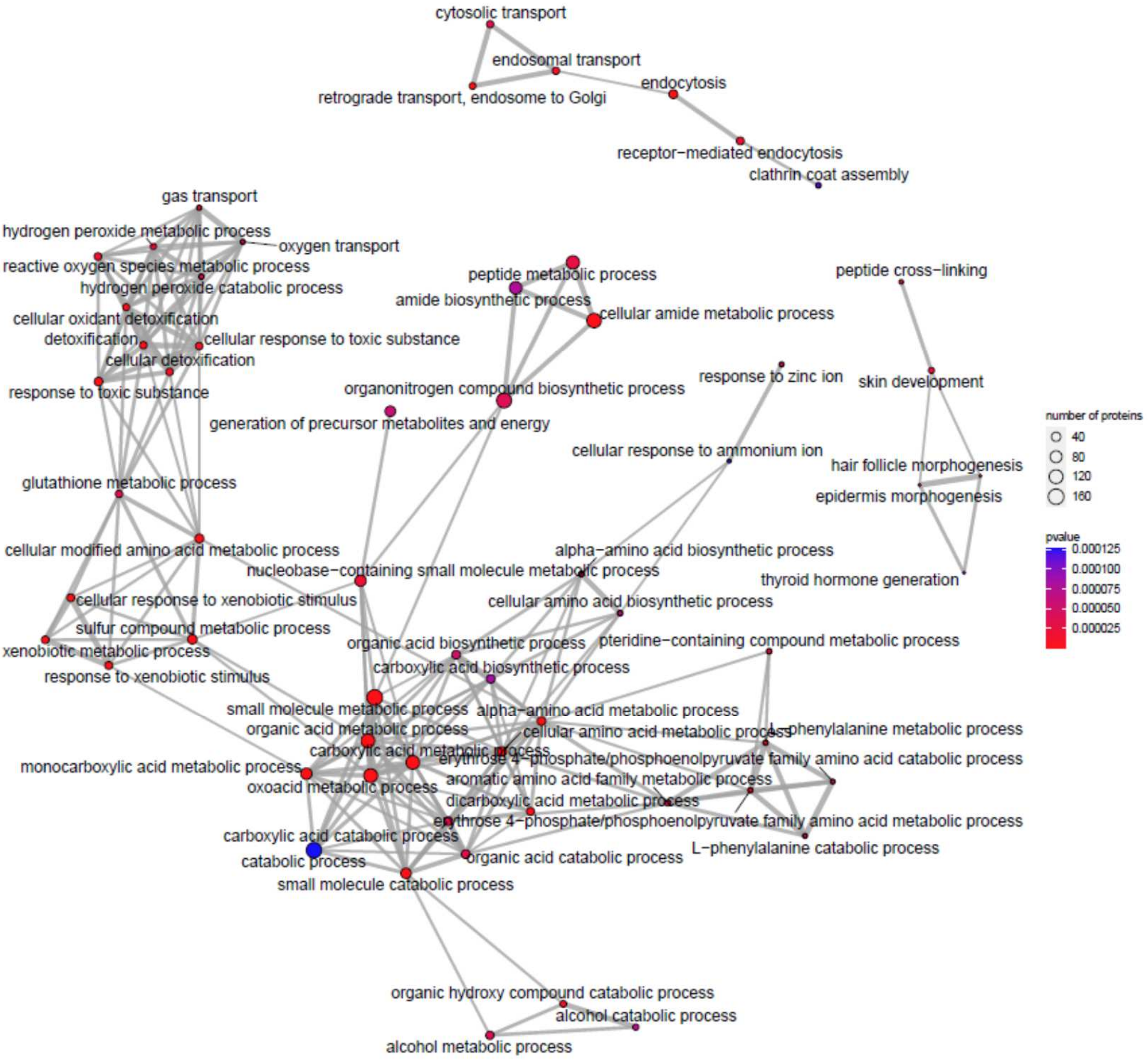

Suplemmentary Figure S1C

(GSEA 60 top CCl_4)

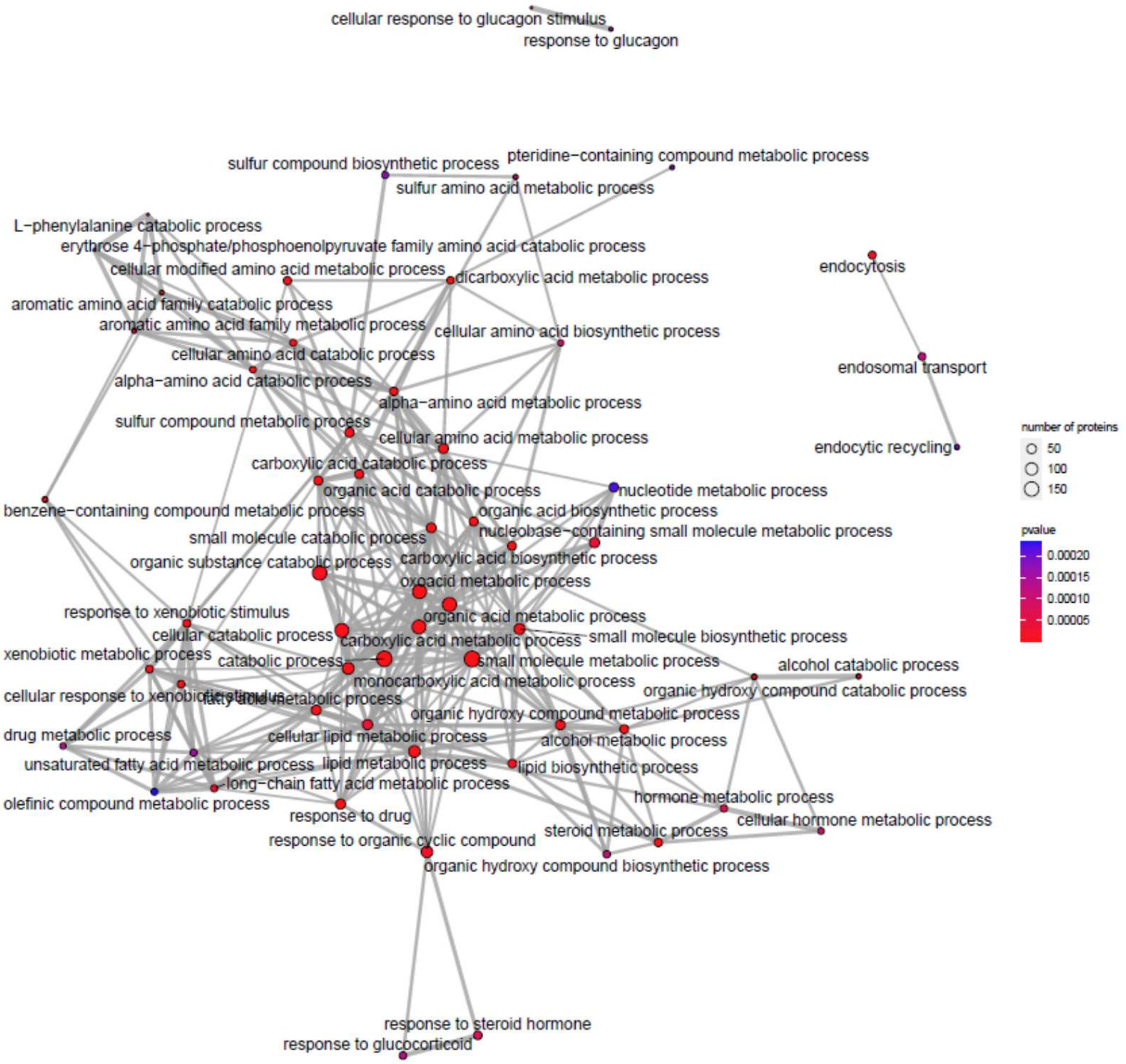

Suplemmentary Figure S2A

(GSEA 5 top BDL)

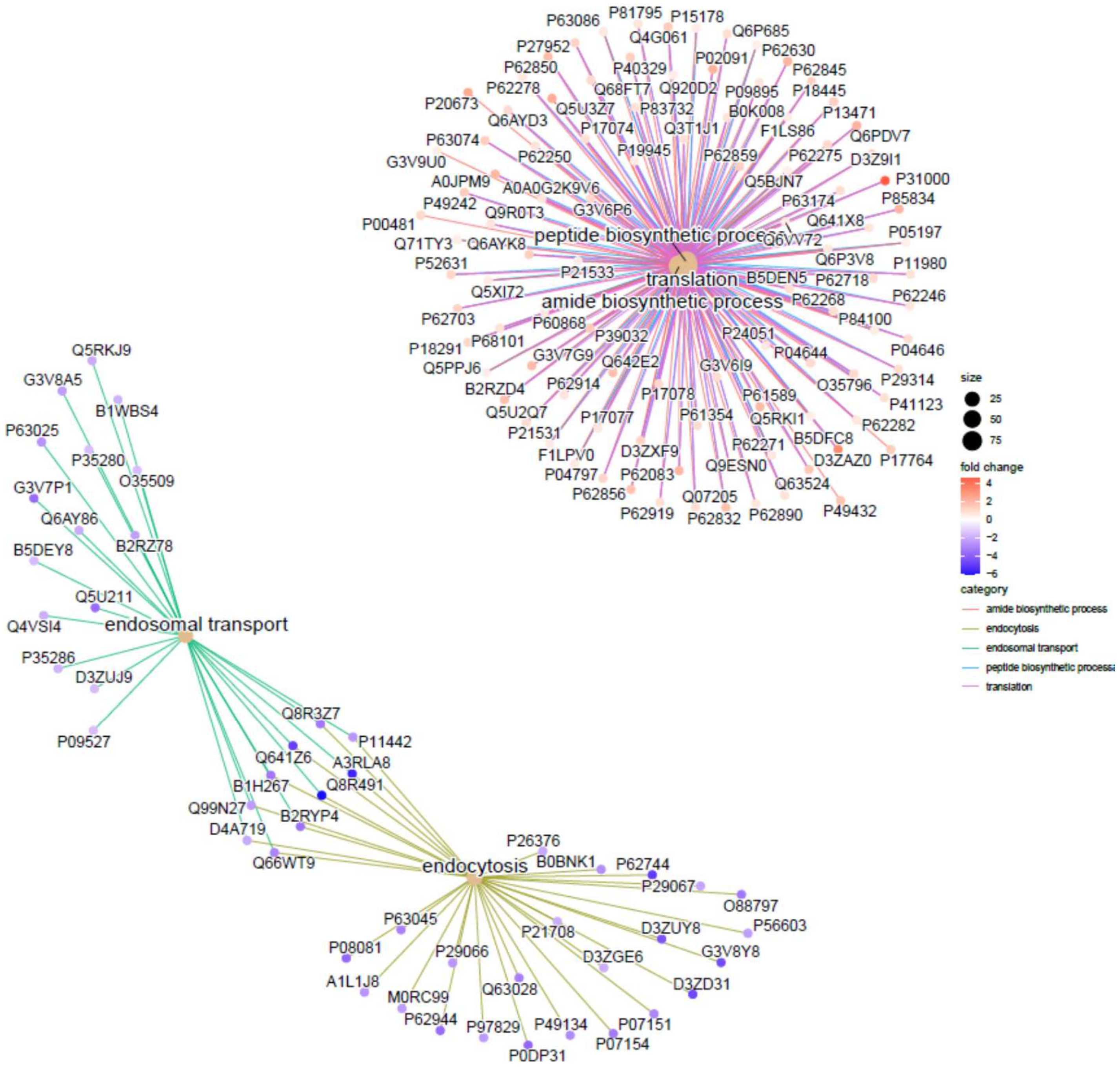

Suplemmentary Figure S2B

(GSEA 5 top HFGFD)

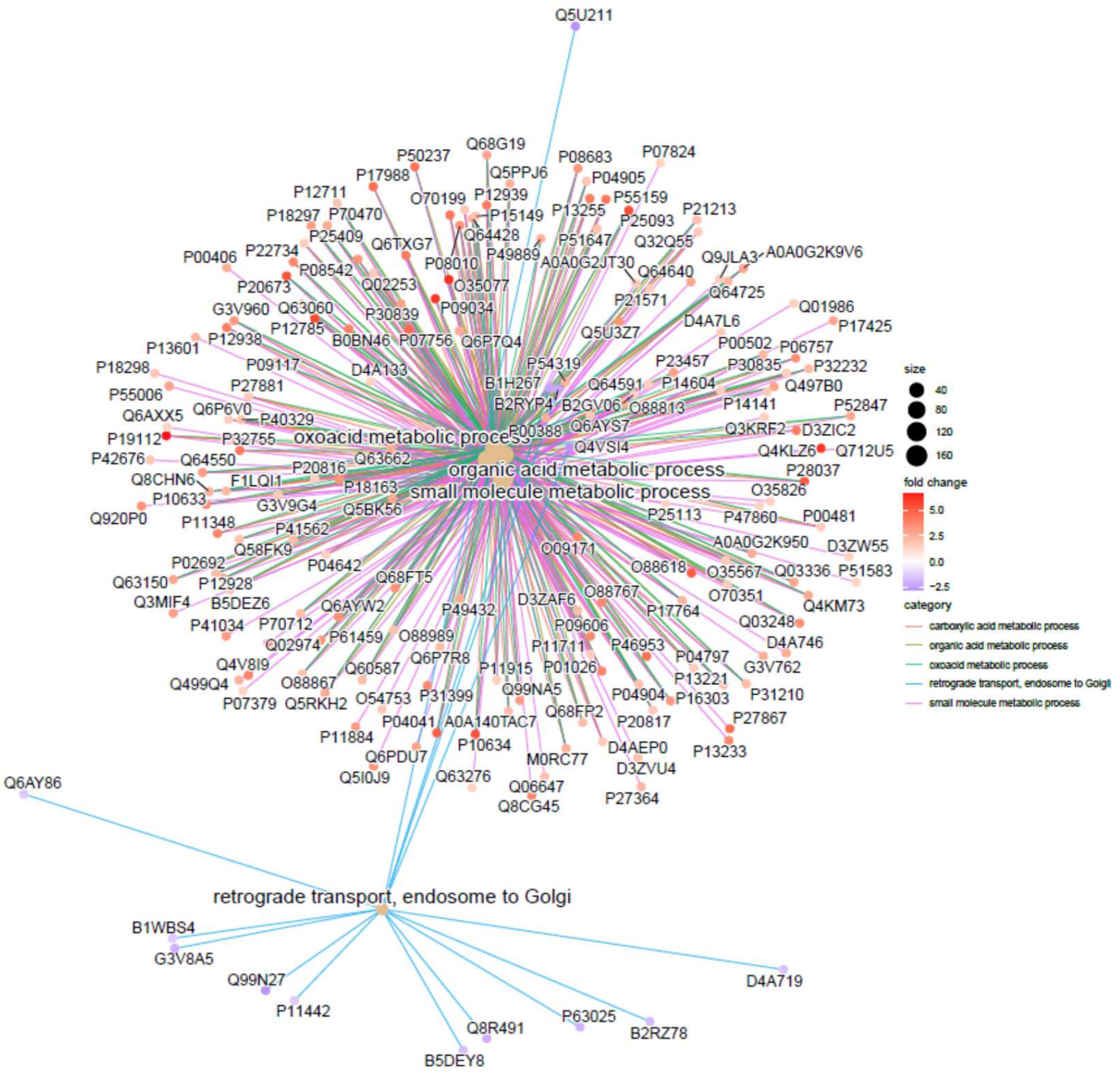

Suplemmentary Figure S2C

(GSEA 5 top CCl_4)

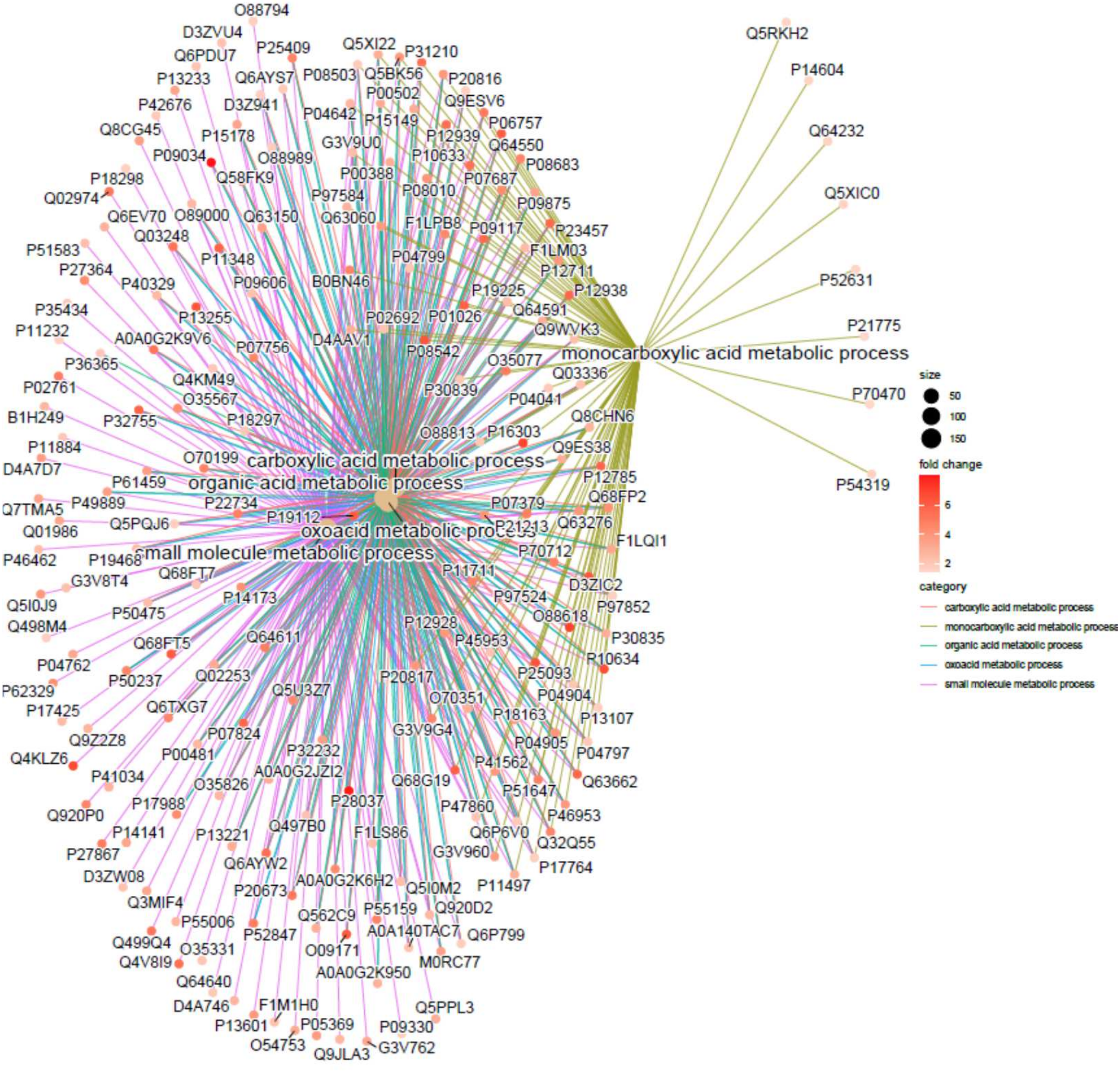

Suplemmentary Figure S3A

(ORA 60 top UP)

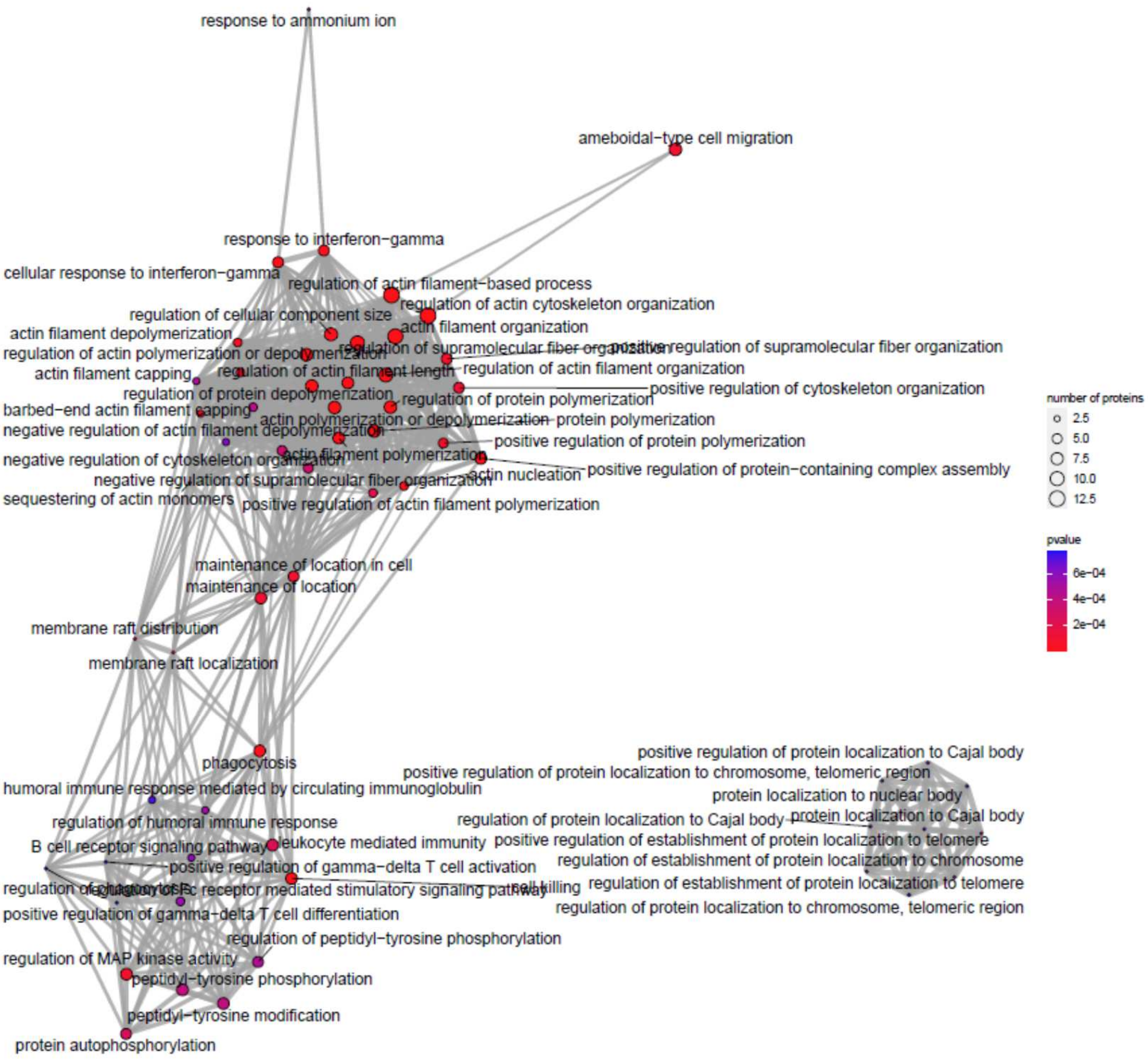

Suplemmentary Figure S3B

(ORA 60 top DOWN)

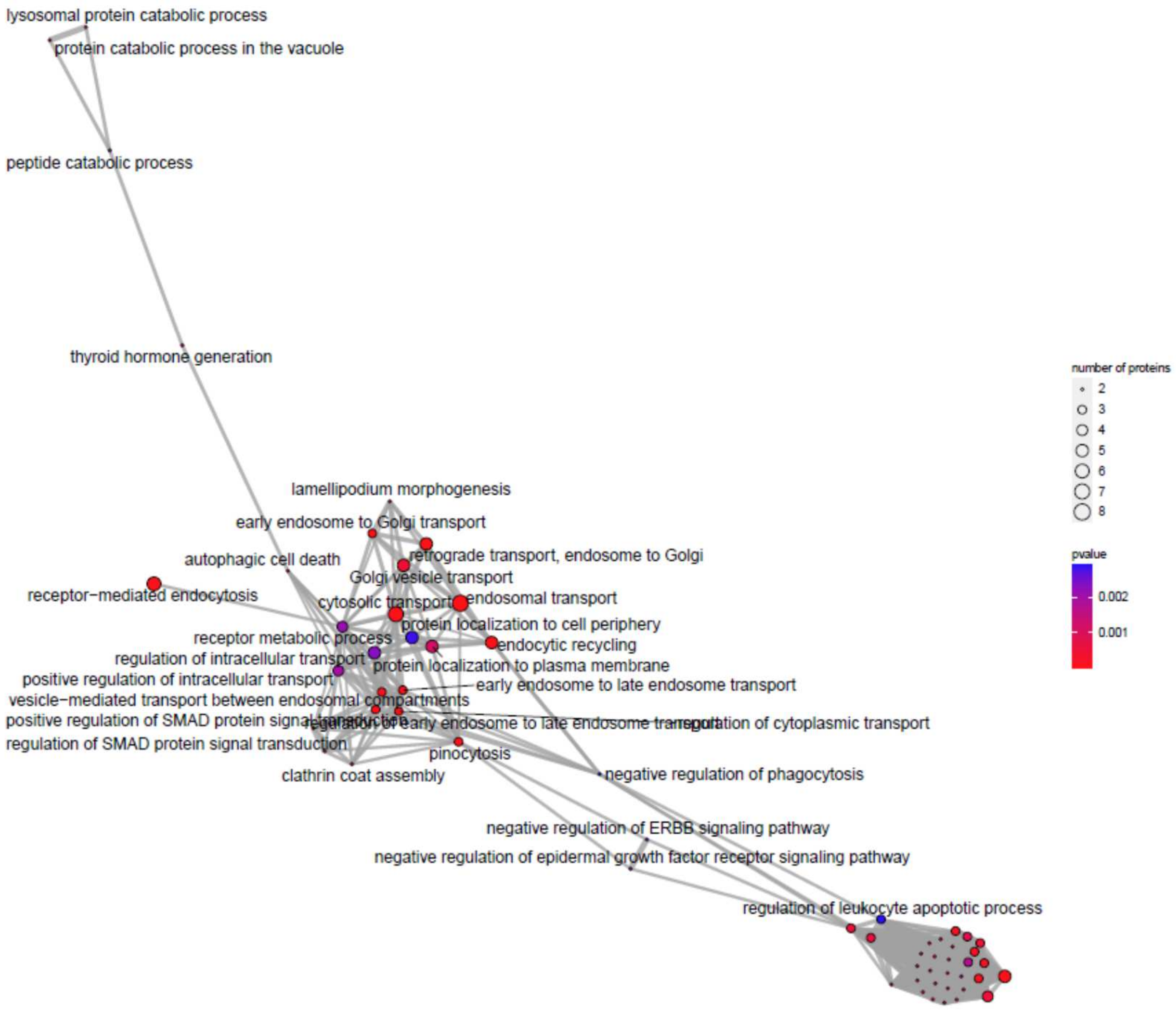

Suplemmentary Figure S4A

(ORA 5 top UP)

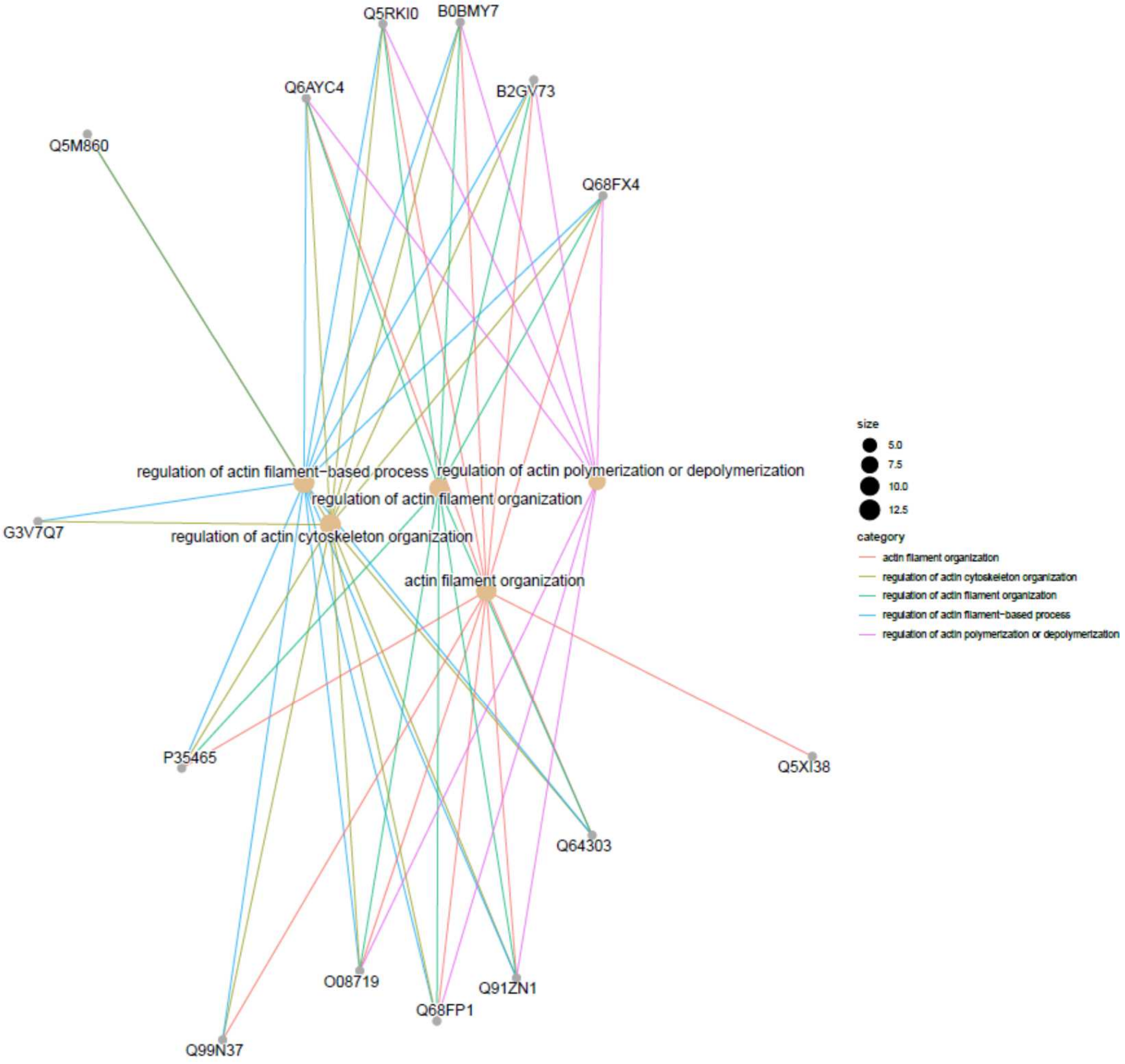

Suplemmentary Figure S4B

(ORA 5 top DOWN)

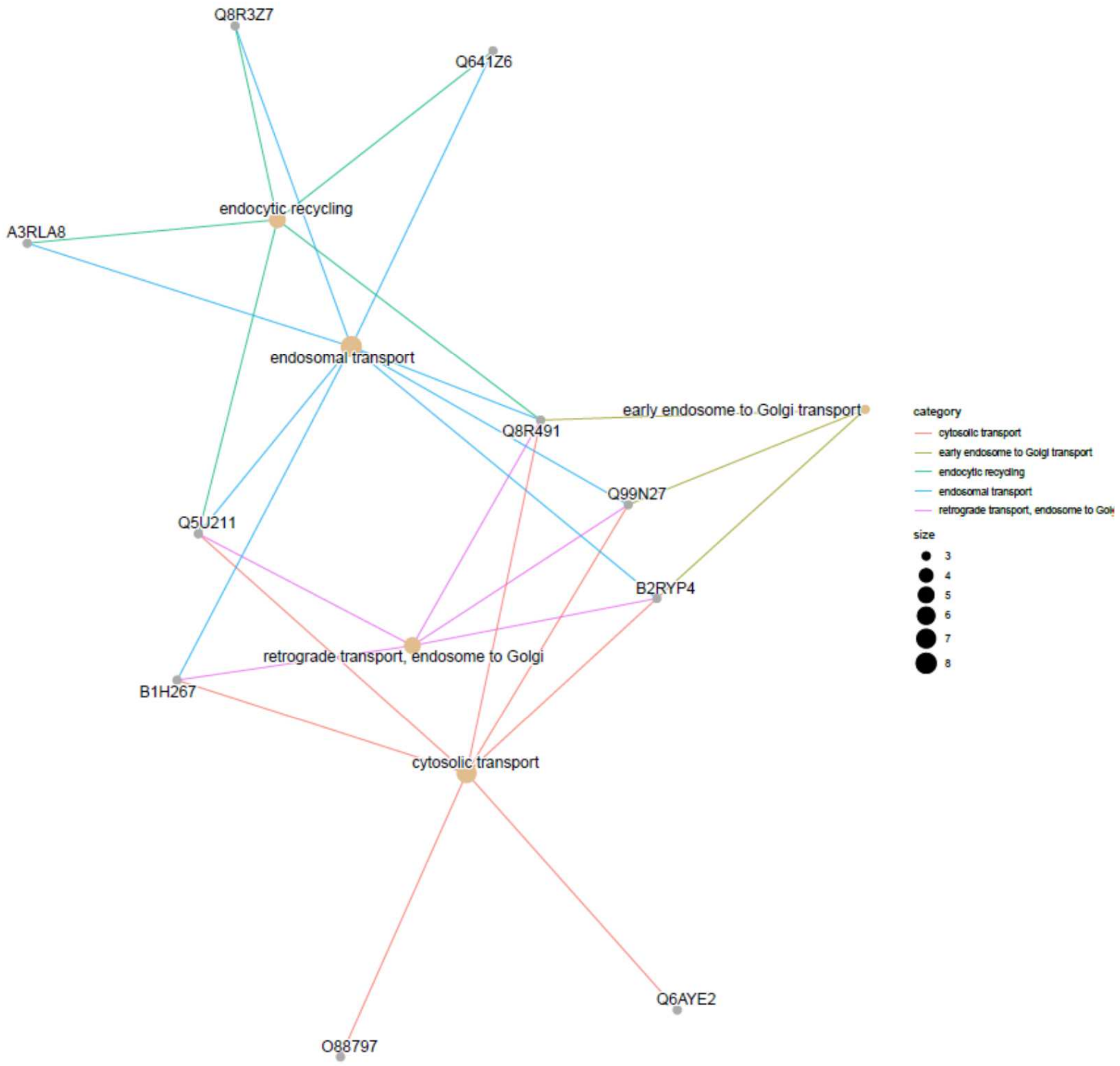

Supplement: Supplementary file 1 [file ijms-24-11904-s001.zip › Supplementary Figures.pdf]
